# Governing E-cigarettes in China: regulatory challenges and strategies for adolescent protection in the post-flavor ban era

**DOI:** 10.3389/fpubh.2026.1813366

**Published:** 2026-03-27

**Authors:** Yu Yao, Luyang Yao, Fei Yang

**Affiliations:** School of Law, China Jiliang University, Hangzhou, China

**Keywords:** electronic cigarettes, flavor ban, public health, regulatory frameworks, tobacco product

## Abstract

China’s e-cigarette governance has entered a complex post-flavor ban era under the “1 + 2 + N” regulatory framework. While this system integrates centralized transaction platforms and mandatory safety standards, critical vulnerabilities remain. Current regulatory asymmetry, characterized by strict e-cigarette flavor prohibitions alongside lenient policies for flavored combustible tobacco, may inadvertently redirect consumption toward traditional smoking products. Meanwhile, the expansion of covert online marketing and illicit social media networks continues to facilitate adolescent access, weakening enforcement effectiveness. Addressing these challenges requires a coordinated policy response. This article proposes extending flavor restrictions to combustible cigarettes to enhance regulatory consistency and raising the legal purchase age with reference to international “Tobacco 21” experience. It further highlights the need to strengthen algorithmic governance to curb hidden digital promotion and to apply nudge-based interventions, such as neutral packaging and graphic warnings, to reduce product appeal. Integrating these supply- and demand-side measures is essential to limit youth initiation, contain illicit market growth, and reinforce long-term public health protection.

## 1 Introduction

In recent years, e-cigarettes have gained global popularity, largely based on claims of harm reduction (1, 2) and smoking cessation (3). However, growing evidence increasingly challenges these assertions. Emerging evidence has suggested that e-cigarette use is associated with adverse oral (4, 5), cardiovascular, and respiratory outcomes (6), as well as heightened viral susceptibility (7). Critically, the extent to which e-cigarettes affect health outcomes varies considerably across product types and use contexts. Key technical and behavioral determinants, such as dual and polytobacco use, device heterogeneity, variations in e-liquid composition, and user behavior (8), differ substantially across product classes and flavor categories. Harm-reduction potential is therefore inherently product-specific and may be functionally undermined in devices or usage patterns that exceed critical risk thresholds. Moreover, unlike established nicotine replacement therapies, e-cigarettes have not been endorsed by the World Health Organization (WHO) as effective cessation tools due to insufficient high-quality clinical evidence (9).

Conversely, some studies have reported associations between e-cigarette use and the subsequent initiation of combustible tobacco use. A systematic review and meta-analysis of prospective studies found that e-cigarette users are more likely to subsequently initiate cigarette smoking compared to non-users (10), though the magnitude of this association varies substantially across studies and the possibility of residual confounding remains debated. While a “gateway effect” has been proposed as one explanation for these associations, it is not the only plausible framework. The common liability framework suggests that shared predispositions, including genetic factors, behavioral disinhibition, and sensitivity to reward, underlie the propensity to use multiple substances, rather than one product causally driving progression to another (11). The diversion hypothesis, by contrast, posits that e-cigarettes may prevent some adolescents from initiating conventional cigarette smoking by satisfying a pre-existing propensity for nicotine use in those who would otherwise have turned to combustible tobacco (12). Regardless of initiation sequence, dual use of e-cigarettes and combustible tobacco represents a distinct pattern of concern in its own right. Evidence suggests that individuals who consume both products experience a higher risk of developing chronic obstructive pulmonary disease than exclusive users (13). According to recent global estimates, approximately 14.7 million adolescents aged 13–15 currently use e-cigarettes, with youth prevalence rates averaging nine times those of adults (14). In addition, exposure to secondhand and thirdhand nicotine aerosol poses long-term developmental risks to pregnant women and children (15), raising ongoing public health concerns.

Given China’s large population and its central role in global e-cigarette manufacturing, the potential public health implications of e-cigarette use warrant close regulatory attention. In response to these concerns, China has implemented a series of regulatory reforms aimed at strengthening oversight of e-cigarette production, distribution, and marketing. A cornerstone of these reforms is the Administrative Measures for Electronic Cigarettes (hereinafter referred to as the *E-Cigarettes Measures*) (16), effective May 1, 2022, which explicitly prohibits the sale of non-tobacco flavored products and open-system devices. Furthermore, these measures prioritize youth protection by banning sales to minors and prohibiting all online sales and advertising. Available evidence indicates that these interventions have produced an initial regulatory effect among adolescents. In 2022, adolescents accounted for approximately 30% of China’s e-cigarette users, and the ever-use rate among middle school students reached 16.1% in 2021. One year after the implementation of the E-Cigarettes Measures, this figure declined to 13.5% in 2023 (17). Consistently, the current e-cigarette use rate among this demographic fell to 2.4% in 2023, representing a decrease of 1.2 percentage points compared to 2021 (18).

Despite these gains, the post-flavor ban landscape presents new regulatory challenges. A persistent gap remains between regulatory intent and market practice, reflected in the proliferation of illegal products, the circumvention of flavor restrictions, and the shift of sales toward decentralized, privately mediated social media channels (19). Although e-cigarette advertising is formally prohibited, covert promotional activities continue to proliferate online, relying on subtle and adaptive digital strategies to evade detection. Meanwhile, existing regulatory measures inadequately address the risk of substitution from e-cigarettes to combustible tobacco. Against this backdrop, this paper examines the practical dilemmas of e-cigarette governance in China’s post-flavor ban era and proposes targeted regulatory strategies aimed at strengthening youth protection.

## 2 China’s e-cigarette regulatory system

### 2.1 The “1 + 2 + N” regulatory framework

China’s e-cigarette regulatory system can be summarized as the “1 + 2 + N” model, which is structured as follows:

The “1” refers to the 2021 revised *Regulations for the Implementation of the Tobacco Monopoly Law of the People’s Republic of China* (20). This primary administrative regulation mandates that e-cigarettes and other next-generation tobacco products be regulated by reference to the relevant provisions governing traditional cigarettes.

The “2” comprises the *E-Cigarettes Measures* and the *Mandatory National Standards for E-Cigarettes* (GB 41700-2022) (hereinafter referred to as *the E-Cigarettes Standards*) (21). The *E-Cigarettes Measures* stipulate the requirements for the production, sale, and import–export of e-cigarettes. Meanwhile, the *E-Cigarettes Standards* provides unified specifications for materials, technical requirements, and other safety parameters of e-cigarette products.

The “N” refers to a multitude of legally binding regulatory documents that facilitate operational oversight. These include specific protocols for product identification and testing, technical reviews, quality supervision, traceability, packaging and labeling, logistics, and the industry credit system. Together, these elements constitute a hierarchical and multi-dimensional framework for the specific implementation of e-cigarette regulation in China.

### 2.2 Key regulatory instruments

The *E-Cigarettes Standards* define e-cigarettes as electronic delivery systems used to generate aerosols for human inhalation, while the *E-Cigarettes Measures* further categorize these products into cartridges, smoking sets, and integrated products sold as a combination of both. Independent of nicotine content or combustion, this definition extends regulatory oversight to individual product components. This inclusive approach grants the framework significant flexibility to address diverse e-cigarette forms. Consequently, the following instruments ensure compliance across production, distribution, and advertising (Table 1).

**Table 1:** Primary regulatory instruments for e-cigarettes in China.

Regulatory instrument	Key descriptions	Legal sources
Traceability & trading platform	All e-cigarette transactions must be conducted exclusively through the “Unified National E-cigarette Transaction Management Platform,” prohibiting sales via any other information networks. Additionally, products must display standardized QR codes on all packaging to enable mandatory traceability across production and wholesale stages.	Articles 19 and 23 of the E-Cigarettes Measures; Article 5 of the Detailed Rules for the Implementation of E-cigarette Product Packaging (50)
Flavor bans	Prohibits all characteristic flavors except tobacco. Ingredients must strictly adhere to a “positive list” of 101 permitted additives.	Article 26 of the E-Cigarettes Measures; Section 4.1.3.1 of the E-Cigarettes Standards
Sales age limit	Strict prohibition on sales to individuals under age 18.	Article 22 of the E-Cigarettes Measures
Advertising restrictions	Bans tobacco advertisements in mass media, public spaces, and transport. All tobacco advertising targeting minors is prohibited.	Article 22 of the Advertising Law of the People’s Republic of China (51)
Packaging and health warnings	The color contrast between background and warning text must satisfy ∆ $E_{\mathrm{ab}}$ ≥40. Packaging must feature health warnings of the mandated size and is prohibited from using misleading language or flavor descriptors appealing to minors.	Articles 3 and 9 of the Regulations on E-cigarette Warning Labels (52); Article 22 of the E-cigarette Measures
Consumption tax	A tax rate of 36% is applied at the production and import stages, while a rate of 11% is applied at the wholesale stage.	Announcement on the Collection of Consumption Tax on E-cigarettes (53)

## 3 Regulatory challenges in e-cigarette governance

### 3.1 Unintended consequences of the flavor ban

China’s flavor ban currently faces risks arising from regulatory asymmetry. While e-cigarette flavors are strictly prohibited, comparable restrictions do not apply to higher-risk flavored combustibles like capsule cigarettes. Without a clear justification, this disparity may undermine the perceived legitimacy of the flavor ban and inadvertently redirect consumer demand toward the combustible tobacco market.

Empirical research on American youth suggests that following the introduction of flavor restrictions, 12.5% of exclusive e-cigarette users increased combustible tobacco use, 7.8% switched to it entirely; and 38.6% of dual users increased cigarette consumption (22), confirming the existence of a substitution effect. Although longitudinal tracking data in China remain limited, a qualitative analysis of mainstream news reports before and after the implementation of the flavor ban indicates that fewer than 30% of surveyed e-cigarette users accepted the national standard tobacco flavor. Common responses included hoarding flavored products, seeking flavored items through informal or illegal channels, or returning to traditional cigarettes (23), reflecting a substitution pattern similar to that observed in other jurisdictions.

This risk is amplified by the expansion of flavored combustible cigarettes in China. Between 2010 and 2020, the annual growth rate of capsule cigarettes in China reached 73.6% (24), underscoring the role of flavor as a central market strategy for the tobacco industry. Under such asymmetrical regulation, the e-cigarette flavor ban may displace demand into combustible markets or unregulated channels, thereby generating new public health risks and increasing regulatory enforcement costs.

### 3.2 Expansion of hidden online communication and trading networks

The gap between strict flavor prohibitions and persistent consumer demand has diverted substantial volumes of e-cigarette transactions into illegal markets (25). Although China has established a centralized monopoly trading platform to supervise lawful distribution, this mechanism is ineffective against unlicensed e-cigarettes that fall outside regulatory oversight at the production stage. Consequently, distribution monitoring remains structurally limited. A survey of Shenzhen middle school students found that since the implementation of the Administrative Measures, 85.71% of respondents reported no added difficulty in obtaining e-cigarettes (26).

In practice, online vendors frequently evade keyword controls by avoiding terms such as “e-cigarette,” instead using suggestive brand names, generic descriptors like “atomizer,” or misleading images. This digital evasion is reinforced by a strategic migration from physical storefronts to private online domains. Field investigations in Shanghai and Chengdu revealed that 26% of physical stores provided QR codes redirecting consumers to private WeChat accounts to complete transactions (27). Concurrently, the modality of promotion on mainstream social media has evolved. On platforms such as Weibo and Xiaohongshu, e-cigarette promotion often appears as “hidden advertising” embedded in lifestyle content, blurring the line between personal expression and commercial marketing. Comment sections have likewise become high-risk spaces for illicit promotion, with some accounts using embedded links or mini-programs as concealed gateways to sales pages (28, 29). A U.S. longitudinal cohort study further demonstrates the behavioral risks of online promotion. Among adolescents with no prior e-cigarette use at baseline, 9.6% initiated use by follow-up. Baseline exposure to e-cigarette advertising on Facebook was associated with higher odds of subsequent use (OR = 2.20, 95% CI 1.37–3.52), even after controlling for demographics and baseline smoking (30). Because such transactions often bypass age-verification mechanisms, adolescents can more easily obtain illegal products with unknown ingredients (23), exacerbating risks to public health and market order.

## 4 Discussion

Before elaborating on specific regulatory recommendations, it is worth noting that the measures discussed in this paper are grounded in China’s specific legal, institutional, and cultural context and may not be directly transferable to other jurisdictions. Different governance systems operate under distinct constitutional arrangements, administrative capacities, and public health priorities, meaning that comparable measures may require alternative legislative pathways or voluntary industry frameworks elsewhere. Cultural dimensions further shape the feasibility and reception of regulatory instruments, as norms surrounding individual autonomy, community responsibility, and digital engagement vary considerably across societies, and interventions calibrated for one context may prove misaligned in another. Even within China, the expansion of these stringent measures faces domestic impediments. Informal social commerce networks have proved remarkably agile in evading platform-level controls, enforcement capacity varies considerably across localities, and entrenched industry interests continue to complicate regulatory implementation. These considerations do not diminish the public health imperative to regulate e-cigarettes, but they do underscore the need for contextually sensitive policy design. The recommendations herein should therefore be understood as proposals developed within China’s regulatory environment, with the recognition that adaptation would be necessary for application in other settings.

### 4.1 Bridging the regulatory gap: expanding flavor bans and raising the age limits

At the legislative level, China should address regulatory asymmetry in flavor restrictions between e-cigarettes and combustible cigarettes. Existing evidence indicates that banning flavors only in e-cigarettes may induce some users to substitute with combustible cigarettes (31). By contrast, comprehensive flavor bans covering both e-cigarettes and combustible cigarettes produce the largest increase in complete tobacco cessation, although they may be accompanied by a limited rebound in combustible cigarette use (32). As of 2024, over 50 jurisdictions worldwide have prohibited or are considering prohibitions on all or partially flavored tobacco products (33). Under the precautionary principle, China should extend the current e-cigarette flavor ban to combustible cigarettes. Given that immediate, comprehensive restrictions on prevalent flavored products may encounter both industrial resistance and deep-seated consumption patterns, a phased reduction in production quotas offers a pragmatic transitional path.

However, acknowledging that this phased implementation leaves a temporary window of availability, China should concurrently raise the minimum purchase age for all tobacco products. This measure acts as an immediate defensive strategy to mitigate early exposure and addiction risks among adolescents and young adults while the comprehensive flavor ban is progressively enforced. This approach is supported by behavioral evidence. A cross-sectional analysis of Wave 1 data from the Population Assessment of Tobacco and Health (PATH) Study in the United States shows that 81.0% of youth aged 12–17 and 86% of young adults aged 18–24 who had ever used tobacco reported flavored products as their first tobacco experience (34). Raising the minimum sales age for all tobacco products to 21 has been associated with declines in both cigarette and e-cigarette use among adolescents (35, 36). A Japanese study further found that individuals who initiate smoking before age 20 exhibit significantly lower cessation success rates than those who begin at age 20 or older (37), indicating that delayed initiation improves long-term cessation outcomes. Consistent with this evidence, the United States raised the federal tobacco purchase age to 21 in 2019 under the “Tobacco 21” policy, after which smoking prevalence dropped by an estimated 12% and related mortality by 10% (38). Although these effects were primarily observed for combustible cigarettes, the policy also targets adolescent e-cigarette use and has been widely adopted by U.S. states following the surge in youth vaping in 2018. Given the comparable addiction risks of e-cigarettes and combustible cigarettes (39), age-based restrictions should apply equally to both product categories. Accordingly, this paper recommends that China uniformly raise the legal purchase age for all tobacco products from 18 to 21, thereby reducing tobacco exposure at its point of entry.

Neuroscientific research indicates that the prefrontal cortex, which governs executive function and impulse control, does not fully mature until approximately age 25 (40), suggesting that individuals aged 21–25 remain in a sensitive developmental period. Raising the sale age limit, as a paternalistic intervention, must adhere to the principle of proportionality. Setting the threshold at 21 targets the highest-risk group while maintaining a reasonable balance between public health protection and individual autonomy, reflecting a soft-paternalistic approach. Within this framework, flavor bans complement age restrictions by offering additional protection to young adults aged 21–25, together forming a layered prevention strategy. However, a 2023 U.S. survey on awareness of the Tobacco 21 policy found that over one-third of respondents were unaware of the increased purchasing age (41). Accordingly, if similar measures are adopted in China, regulatory reforms should be accompanied by enhanced public education and compliance outreach to ensure effective implementation.

### 4.2 Strengthening platform responsibility and algorithmic governance

Third-party e-commerce platforms should shift from passive content blocking to proactive transaction prevention in regulating e-cigarette sales. Platforms need to continuously upgrade multimodal intelligent recognition technologies to detect and block variant keywords (especially brand names), train domain-specific semantic models to capture obscure expressions, and apply computer vision for image-text matching to intercept e-cigarette-shaped images and suggestive descriptions. Controls on external links should also be strengthened to prevent redirection to private messaging apps for illicit deals. In parallel, platforms should improve complaint and reporting mechanisms, broaden social supervision channels, and improve response speed and accuracy.

Governance of e-cigarette information on social media platforms is more complex and requires precise targeting of implicit marketing while preserving user experience. For minors, strict age-verification mechanisms comparable to anti-addiction systems in video games should be adopted, including restricting keyword searches on minor accounts, algorithmically prioritizing health education content, and blocking posts describing e-cigarette use experiences. For adult users, regulation should focus on distinguishing commercial promotion from personal expression. Algorithms should be developed to automatically identify features such as high-frequency brand names and marketing terminology and to limit traffic to suspected promotional content, accompanied by robust appeal and correction mechanisms. For ambiguous content, mandatory warning labels such as “Smoking is harmful to health” should be applied.

At the technical level, platforms can strengthen governance by targeting recurring marketing themes, especially conceptual flavors conveyed through indirect descriptors, such as terms implying fruit or dessert tastes (e.g., “Tropical” implying pineapple). This approach requires the establishment and dynamic updating of e-cigarette concept databases, together with more refined marketing content detection techniques (42). Existing research provides a methodological basis by organizing e-liquids into major categories such as tobacco and fruit and further dividing them into around 90 subcategories, such as berries and citrus, within a flavor wheel (43). This hierarchical framework offers a standardized vocabulary for classifying marketing descriptions. Building on it, automated image recognition, data extraction, and large language models can be applied to map marketing descriptors onto the flavor wheel and construct a semantic database of e-cigarette descriptions. In light of cultural differences in flavor expression, Chinese platforms should further identify localized descriptive terms and develop corresponding semantic mappings to enhance regulatory precision.

Government authorities should incentivize platforms to internalize compliance and governance costs, rather than rely solely on administrative orders or subsidies. This can be achieved by clarifying statutory responsibilities, strengthening enforcement and penalties, providing technical guidance and shared resources, and offering incentives such as preferential compliance ratings, thereby supporting sustainable public health goals.

### 4.3 Refining packaging design: a nudge-based approach

Compared with strict regulation, tobacco packaging design functions as a milder complementary instrument that can guide consumers away from e-cigarette use through nudge theory. Nudge theory seeks to correct irrational behavior through subtle, non-coercive interventions while preserving individual choice, thereby facilitating behavior change (44). Tobacco packaging primarily consists of background, warning labels (text and images), and implicit thematic marketing. Current Chinese regulations mainly address text–background contrast, warning label size and color, and language restrictions.

Some studies propose positive nudges through differentiated packaging to direct consumers toward “lower-harm” products, such as using color intensity to signal nicotine levels or emotive icons to indicate harm (45). However, such approaches often complicate choice architecture, increasing information load and decision failure. Moreover, vivid background colors are closely associated with thematic marketing, for example orange evoking fruit flavors (42), which may circumvent language restrictions by conveying sensory cues that enhance product appeal and mislead consumers.

Accordingly, packaging nudges should shift toward direct negative interventions. This includes adopting uniform neutral background colors, such as black or white, to sever visual links to marketing themes, and using high-visibility colors like yellow or orange for warning text (46, 47) to generate strong contrast and draw attention. Evidence further indicates that text-only warnings currently used in China have limited deterrent effects, whereas combined graphic warnings depicting internal pathology or affected individuals are more effective than single-modality warnings (48, 49).

In summary, future tobacco packaging regulation in China should adopt an integrated design combining neutral backgrounds, high-contrast warning text, and graphic warning labels explicitly depicting tobacco-related diseases. Such a model delivers stronger negative nudges and enhances public risk awareness in coordination with other regulatory measures.

## 5 Conclusion

Aligning flavor restrictions with increased age limits, supported by strengthened platform governance and evidence-based packaging design, is essential to reduce youth vaping initiation and contain illicit market expansion. Although the absence of long-term domestic longitudinal data remains a key limitation, these measures establish a necessary foundation for public health protection. Future regulatory adjustments should remain evidence-based, with particular emphasis on sustained research into behavioral changes among Chinese adolescents to ensure the long-term effectiveness of tobacco control policies.

## Data Availability

The original contributions presented in the study are included in the article/supplementary material, further inquiries can be directed to the corresponding author.
